# Comparative proteomic analysis of mustard lung as a complicated disease using systems biology approach

**DOI:** 10.1186/s12890-022-02240-3

**Published:** 2022-11-23

**Authors:** Shahram Parvin, Masoud Arabfard, Ali Ghazvini, Mostafa Ghanei, Ali Najafi

**Affiliations:** 1 Education Office, Pasteur Institute of Iran, Tehran, Iran; 2 Chemical Injuries Research Center, Systems Biology and Poisonings Institute, Baqiyatallah University of Medical Sciences, Tehran, Iran; 3 Molecular Biology Research Center, Systems Biology and Poisonings Institute, Baqiyatallah University of Medical Sciences, Tehran, Iran

**Keywords:** Mustard lung, Systems biology, Proteomics, Enrichment analysis

## Abstract

During Iraq-Iran conflict, chemical weapons, particularly SM gas, were used numerous times, whose aftereffects are still present. This study aimed to compare serum proteome in the chronic ML (n = 10) and HC (n = 10). TMT label-based quantitative proteomics was used to examine serums from two groups. Among total significant proteins, 14 proteins were upregulated (log_2_ ≥ FC 0.5, *p* 0.05), and 6 proteins were downregulated (log_2_ ≤ FC − 0.5, *p* 0.05). By helping PPI network, and EA, 11 main pathways connected to significantly different protein expression levels were discovered, including inflammatory and cell adhesion signaling pathways. It may be deduced that the wounded organs of exposed individuals experience poor repair cycles of cell degeneration and regeneration because certain repair signals were elevated while other structural and adhesion molecules were downregulated. The systems biology approach can help enhance our basic knowledge of biological processes, and contribute to a deeper understanding of pathophysiological mechanisms, as well as the identification of potential biomarkers of disease.

**Supplementary Information:**

The online version contains supplementary material available at 10.1186/s12890-022-02240-3.

## Introduction

Sulfur mustard (SM), bis (2-chloroethyl) sulfide, known as "mustard gas", is a type of chemical warfare agent with destructive effects on the lung, eye, skin, cardiovascular and digestive systems of humans [1, 2]. The lung is one of the body's organs that is vulnerable to injury. There is evidence that SM may produce a number of lung problems, including sub-epithelial fibrosis, chronic bronchitis, and chronic pulmonary obstruction, around 40 years after exposure (COPD). Patients exposed to SM have clinical symptoms that are comparable to those of COPD. Depending on the severity of pulmonary complications caused by SM, the patient’s exposure to SM is divided into three categories: mild, moderate, and severe [3]. Considering the uncertainty of exact pathophysiology of lung exposed to SM, several mechanisms in the lungs were proposed, including oxidant-antioxidant imbalance, immune system irregularities (systemic innate and adaptive immune cell alterations including: NK cells, CD4+ and CD8+ T cells) and increased inflammatory mediators, such as cytokines in the lung tissue, Broncho alveolar lavage (BAL), as well as serum [4–11]. Recent studies on the immunopathogenesis of chronic lung diseases, such as COPD, which is very similar to the disease under study, have shown an association with changes in Th17/Treg [12, 13].

Unfortunately, the exact mechanism of the effects of SM toxicity on the lungs over a long period of time is not known. Consequently, based on the explanations of the clinical symptoms of these patients, it is required to conduct more comprehensive research to know more about the symptoms of the mustard lung (ML) in the chronic phase of the disease. Treatment and diagnosis of people with ML, compared to healthy controls (HC) with a systems biology approach, are helpful in more accurate diagnosis of this disease. Regarding the systemic nature of this disease, to better understand the biological mechanisms of the disease, systems biology approach is needed. Proteomics studies, especially labeling method by Tandem Mass Tag® (TMT®) coupled with LC–MS/MS technology, are one of the best choices to identify and quantify the proteins in ML and HC groups which can be used in serum samples [14–16]. After the filtration of the proteome data and obtaining the differential abundance proteins (DAPs), the specific candidate proteins of this disease were identified to analyze the data resulting from the proteomics study. Based on enrichment analysis (EA), along with other bioinformatics analyses for protein identification and quantification candidates. EA is a powerful tool to examine the relationship between phenotype disease and a group of genes, as well as proteins. The selection and input criteria for EA are based on quantitative expression data for each gene or protein [17].

Gene ontology (GO) deals with the characteristics and functions of genes and genetic products. GO generally covers three domains (cellular component, molecular function, and biological process) [18]. The coordination of intracellular mechanisms is in terms of molecular interactions. Molecular interactions, which are continuous, cause the molecular pathways of cells to regulate and execute complex processes [18]. Among biological samples, the serum is a part of the blood which contains proteins, and other biological molecules which represent the body's biological mechanisms which is rich in biological information; using blood as the source of biomarkers can be a less invasive and less expensive method [19–22]. Nowadays, the biomarkers can be obtained via technologies, such as proteomics [23–25]. This study aimed to find proteome patterns in chronic ML. The reason to choose men is that the mechanism of SM effect on women may have different pathophysiology [26, 27], and due to the grouping of people included and the lack of access to the statistical population of women. In this regard, via systems biology approach, this study can be very useful to enhance our basic knowledge of biological processes and a deeper understanding of pathophysiological mechanisms of disease, as well as in clinical research to identify potential biomarkers of disease plus drug targets.

## Materials and methods

### Study population and design

**Fig. 1:**
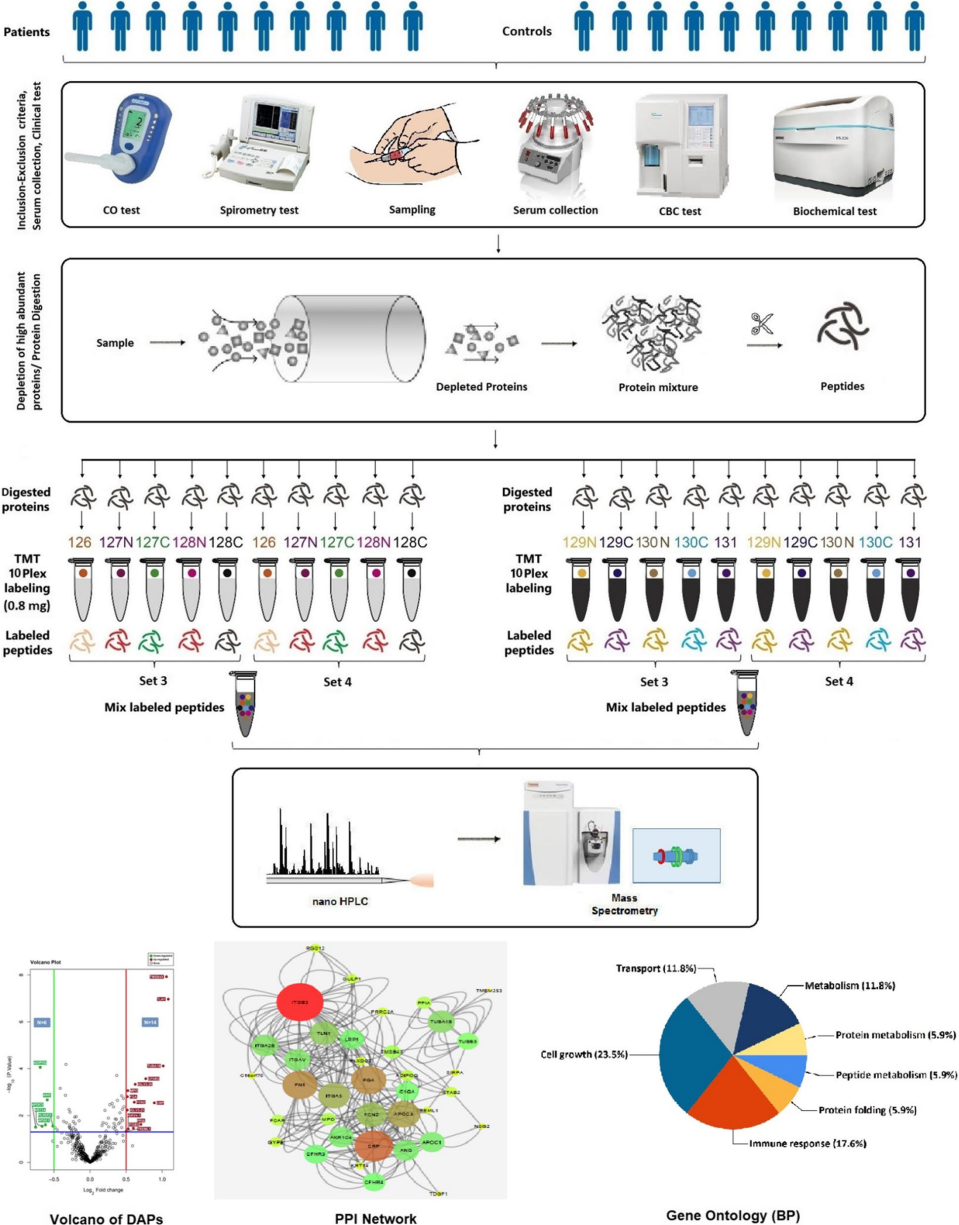
Schematic representation of the study design, proteomic work flow and bioinformatics analysis

This study was conducted with an ethical code at Baqiyatallah University of Medical Sciences (IR.BMSU.REC.1395.381). The participants were categorized due to degree of lung disease before being sampled based on American Thoracic Society (ATS) categorization. All volunteers were asked to participate in routine clinical tests, such as pulmonary function testing (PFT) (Multi-Functional Spirometer HI-801), carbon monoxide (CO) ( Bedfont piCO™ Smokerlyzer®), as well as hematological (Mindray Bc-3000) and biochemical analysis (Mindray Bs-230) consisting of CBC diff, FBS, lipid profile, Uric acid, AST, ALT Urea, Creatinine, and CRPq. Adult men with ML (n = 10) and HC (n = 10) took part. The inclusion and exclusion criteria of ML and HC groups are mentioned in Table 1. ML and HC would be able to participate in the research once the inclusion and exclusion criteria were checked and adapted for them. The final confirmation of ML disease was done by a specialist in the medical committee on verifying chemical-warfare damaged people. According to committee’s opinion, ML and HC participants were chosen. Schematic representation of study design is presented in Fig. 1.

**Table 1:** Inclusion and exclusion criteria of ML and HC

*Inclusion criteria*
Adult male
Non-smoker
Age between 45 and 60 years
FEV1/FVC > 70%, FEV1 > 80% for HC
55% < FEV1 < 100% for ML
*Exclusion criteria*
Taken Corticosteroids or other specific drugs for 2 weeks
Diabetic, Hypertension, Gout, Asthma and Hyperlipidemia
Surgery in the last 3 months
Lung infection in the last month
Seasonal allergies in the last 6 months
Treated with antibiotics for 2 weeks

ML, Mustard Lung; HC, Healthy Control; FEV1% Pred, Predicted forced expiratory volume in one second; FVE, Forced Vital Capacity

### Sample processing

#### Sample preparation

Blood samples were taken from these subjects to perform the mentioned tests. For this purpose, after filling out the questionnaire and obtaining informed consent, 10 ml of human's whole blood samples from ML and HC groups, which were taken based on age and gender in the Baqiyatallah Hospital. Then, between 8 and 9 o'clock, the blood sample of ML and HC participants was collected as 12-h fasting in a blood clot tube without anticoagulant (BD Vacutainer® Venous Blood Collection). Then, the blood was kept at room temperature (RT) for 10 min, and after clotting, to separate the serum, the samples were centrifuged with a refrigerated centrifuge (2500 g for 10 min) (Sigma 3-30KS). The serums were aliquoted into polyethylene tubes (Eppendorf Tubes®1.5 ml). Then, the serum samples were lyophilized (4 °C for 24 h). The samples were kept in a deep freezer at 80 °C (Thermo Scientific™) until they were used.

Lyophilized samples for proteomics analysis were sent to Australian Proteome Analysis Facility (APAF) laboratory. In brief, lyophilized sample pellets were reconstituted to deplete abundant proteins and washed in phosphate buffer saline buffer (PBS). The samples were subjected to the abundant protein depletion kit (CAT NO: 85165 Thermo Scientific Pierce™). Using protein assay (CAT NO: 23235 Thermo Scientific™), the concentration of proteins in the lyophilized serum samples was determined. Using Dithiothreitol (DTT) and Ioadoacetamid (IAA), disulfide bonds in cysteines were reduced, and their number diminished. Eventually, using trypsin enzyme, the proteins were digested for the sequencing stage.

#### LC–MS/MS analysis

Isobaric Label Reagent TMT-10plex (CAT NO: 90110 Thermo Scientific™) peptide labeling was done based on the manufacturer's instructions for overall procedure. In brief, anhydrous acetonitrile (ACN), hydroxylamine (5%) was used to label each protein sample at RT. A "label check" experiment was performed before pooling the samples to ensure an equal amount of total peptides were pooled from all samples. TMT-labeled peptide samples were pooled at a 1:1 ratio across all samples and vacuum dried after determining the normalization factor. Desalting using C18 solid-phase extraction and vacuum centrifugation to full dryness was used to clean the samples. In the next stage, the samples were fractionated using a high pH reversed-phase kit (CAT NO: 84868 Thermo Scientific™). At this stage, peptides and proteins were separated using a nanoLC system via a nano-LC column. (Halo-C18, 160 Å, 2.7 µm, 100 µm × 20 cm). For MS/MS experiments, the eluent from the column was pumped into the mass spectrometer's ionization chamber (Thermo Scientific™—Q Exactive HF-X™ Hybrid Quadrupole—Orbitrap Mass Spectrometer). Peptide precursors with molecular weights ranging from 350 to 1850 m/z were scanned at 60 k resolution. Higher Energy Collisional Dissociation (HCD) was used to fragment the 20 most energetic ions in the survey scan, with a normalized collision energy of 35 and a precursor isolation of 0.7 m/z. Dynamic exclusion was set to 90 s, and MS/MS scan resolution was set to 60 k.

### Data analysis

Proteome Discoverer™ software (version 2.1, Thermo Fisher Scientific™) was used to search the raw data files for each sample set for data analysis of Orbitrap Fusion data. The data were compared to all *Homo sapiens* sequences retrieved from SwissProt database using the search engines SequestHT (version 2018) and Mascot (version 2.4, Matrix Science, London, UK) (version 2018). The names of proteins were determined, and the gene symbol was obtained from the Uniprot database (http://www.uniprot.org), which contains 95,106 human proteins, including isoforms and unreviewed (*Homo sapiens*). Table 2 reports the parameters for data processing. Using the abundances of the sample controls as the denominators, TMT-10 plex kit calculated the quantitative ratios in two sets.

**Table 2:** Database retrieval parameter

Database	SwissProt database (version 2018)
Enzyme name	Trypsin
Maximum missed cleavage	2
Static modification of cysteine	Carbamidomethylation (C)
Dynamic modifications	TMT 10-plex (N-term, K), Oxidation (M), Deamidated (N, Q), Glu- > pyro-Glu (N-term E), Gln- > pyro-Glu (N-term Q) and Acetyl (Protein N-Terminus)
Precursor mass tolerance	10 ppm
Fragmentation mass tolerance	0.02 Da
FDR and result display filtered	Protein, Peptide and PSM FDR < 1%, Master Proteins

#### Differential abundance proteins (DAPs) analysis

A linear model was used for the statistical analysis of differentially abundant proteins using the *Limma* package, which is a fundamental component of Bioconductor in the R programming environment [28, 29]. Using *Limma* Package (|Log_2_FC|> 0.5, t-test *p* value < 0.05) to find DAPs, a comparison among the data from proteome sequence analysis was done. The normalized protein areas between control and disease samples were compared using *Limma* Package (|Log_2_FC|> 0.5, t-test *p* value < 0.05) in serum samples.

#### Network and enrichment analysis (EA)

Physical relationships among differentially abundant proteins were searched for protein–protein interactions (PPIs) using GeneMANIA tool (https://genemania.org/) to uncover more effective proteins in ML patients compared to HC PPIs network [30].

A famous database, Functional Enrichment analysis tool (FunRich) [31, 32], was used to determine how enriched biological pathways (KEGG) and GO Enrichment in patients compared to controls were related to each other [33, 34]. GO Consortium and KEGG pathway analysis provide functional annotations that support high-throughput data such as proteomes with a system biology approach.

## Results

### Study participants

A total of ten ML and ten HC were recruited. Males made up the entire group of 20 subjects. For ML, the average age was 53.7 years (range 48–60), while for HC, the average age was 47.5 years (range 45–55). Among the participants in the study, people with a BMI < 30.0 were included in the study. Height, weight, BMI index, spirometry indices, and leukocyte count did not significantly vary between the two groups. Only the difference in HRCT values between the two groups among the clinical cases presented in Table 3 was statistically significant; thus, all participants in the ML group had an HRCT value more than 8.0, whereas none in the HC group did. We could reach 20 ML and HC groups out of the 56 participants who were recruited. Serum was taken from the remaining participants and made accessible for proteomic analysis. These descriptive statistics are presented in Table 3.

**Table 3:** Demographic, clinical summary of the ML and HC

	Characteristic	ML (n = 10)	HC (n = 10)	*p* value
Demographic	Age ≤ 55 yr, n(%) [range]	7(70%) [48–60]	9(90%) [45–55]	0.582
	*Height(cm) [range]	173.6 ± 5.1 [165–181]	170.5 ± 3.5 [165–175]	0.13
	*Weight(kg) [range]	80.7 ± 11.5 [68–105]	76.2 ± 9.3 [62–91]	0.35
	*BMI, kg/m^2^	26.8 ± 4.1	26.2 ± 3.6	0.73
HRCT	Air trapping ≥ 8	100.0% (10)	0.0% (0)	< 0.0001
Spirometry	*FEV1% Pred (%)	87.8 ± 11.1	95.4 ± 9.8	0.12
	*FVc(%)	97.2 ± 5.3	98.2 ± 6.3	0.70
	*FEV1/FVc (%)	101.2 ± 9.3	107.5 ± 8.9	0.14
Leukocyte	*Count 1000ul	7.16 ± 1.9	6.54 ± 1.3	0.41

^*^Data are presented as mean ± SD and P value; BMI, Body Mass Index; FEV1% Pred, Predicted forced expiratory volume in one second; ML, Mustard Lung; HC, Healthy Control; FVE, Forced Vital Capacity; HRCT, High-resolution computed tomography

### Proteomic characteristics and identification of DAPs

The high abundant proteins decreased the fibrinogen, 1-acid glycoprotein, 1-antitrypsin, haptoglobin, 2-macroglubulin, IgA, albumin, IgG, apolipoprotein A-I, IgM, apolipoprotein A-II, and transferrin. To study differences in the expression in ML and HC serum samples, TMT labeling LC–MS/MS proteomic approach was used.

**Fig. 2:**
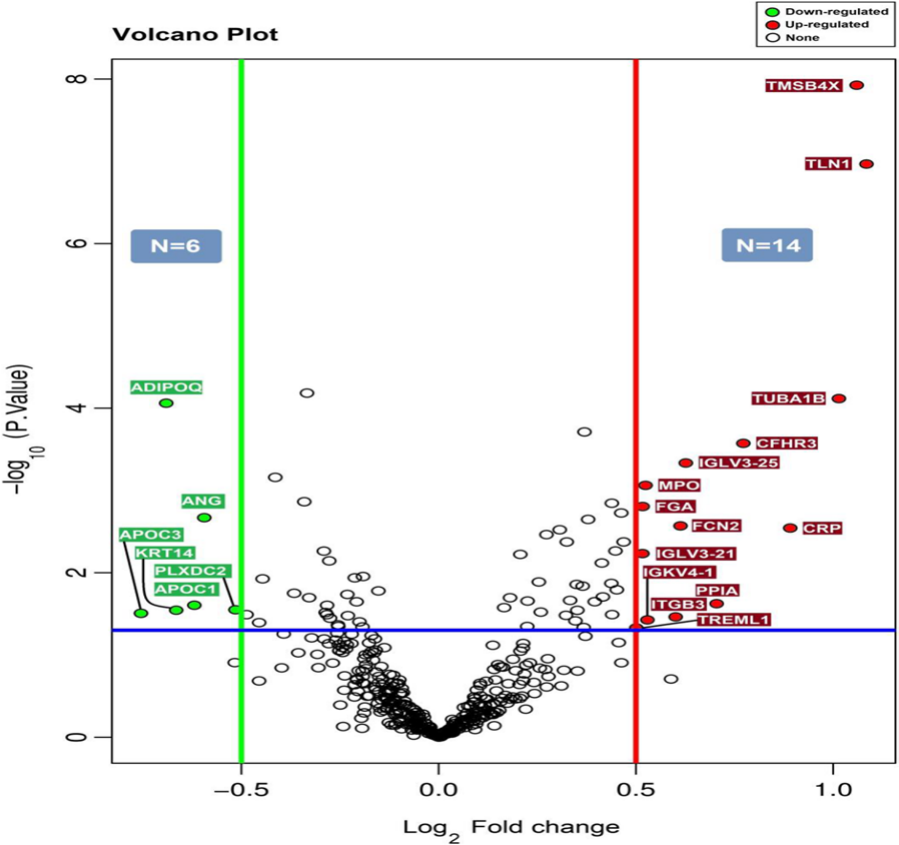
Volcano diagram for significant expression differences proteins of ML versus HC

Based on the sequencing data of the proteome of ML and HC serum sample to find DAPs, overall, 20 proteins were found with significant differential protein expressions. *P* values were calculated from criteria t-test statistics (*p* value < 0.05), (|Log_2_FC|> 0.5) in serum samples, where log_2_FC ≥ 0.5 was upregulated, while log_2_FC ≤ -0.5 was downregulated. A total of 20 proteins for ML versus HC, including 14 upregulated and 6 downregulated proteins (Tables 4, 5), were found as shown in the volcano diagram (Fig. 2). Furthermore, the number of significant and non-significant proteins is given in Additional file 1.

**Table 4:** List of significantly up-regulated proteins level in serum of ML compared HC by t-test

Uniprot accession	Gene symbol	Protein name	FC ^n^	*p* value
P62328	TMSB4X	Thymosin beta-4	2.08	1.19E−08
Q9Y490	TLN1	Talin-1	2.12	1.08E−07
P68363	TUBA1B	Tubulin alpha-1B chain	2.02	7.65E−05
Q02985	CFHR3	Complement factor H-related protein 3	1.70	0.0002
P01717	IGLV3-25	Immunoglobulin lambda variable 3–25	1.54	0.0004
P05164	MPO	Myeloperoxidase	1.43	0.0008
P02671-1	FGA	Fibrinogen alpha chain	1.43	0.0015
Q15485	FCN2	Ficolin-2	1.52	0.0026
P02741-1	CRP	C-reactive protein	1.85	0.0028
P80748	IGLV3-21	Immunoglobulin lambda variable 3–21	1.43	0.0058
P62937	PPIA	Peptidyl-prolyl cis–trans isomerase A	1.62	0.0237
P05106	ITGB3	Integrin beta-3	1.51	0.0344
P01625	IGKV4-1	Immunoglobulin kappa variable 4–1	1.44	0.0374
Q86YW5	TREML1	Trem-like transcript 1 protein	1.41	0.0469

^n^Fold change of case compared controls group

**Table 5:** List of significantly down-regulated proteins level in serum of ML compared HC by t-test

Uniprot accession	Gene symbol	Protein name	FC ^n^	*p* value
Q15848	ADIPOQ	Adiponectin	0.61	8.70E−05
P03950	ANG	Angiogenin	0.66	0.0021
P02654	APOC1	Apolipoprotein C-I	0.65	0.0248
Q6UX71	PLXDC2	Plexin domain-containing protein 2	0.69	0.0282
P02533	KRT14	Keratin, type I cytoskeletal 14	0.63	0.0285
P02656	APOC3	Apolipoprotein C-III	0.59	0.0311

^n^Fold change of case compared controls group

### Gene ontology (GO) and pathway analysis

**Fig. 3:**
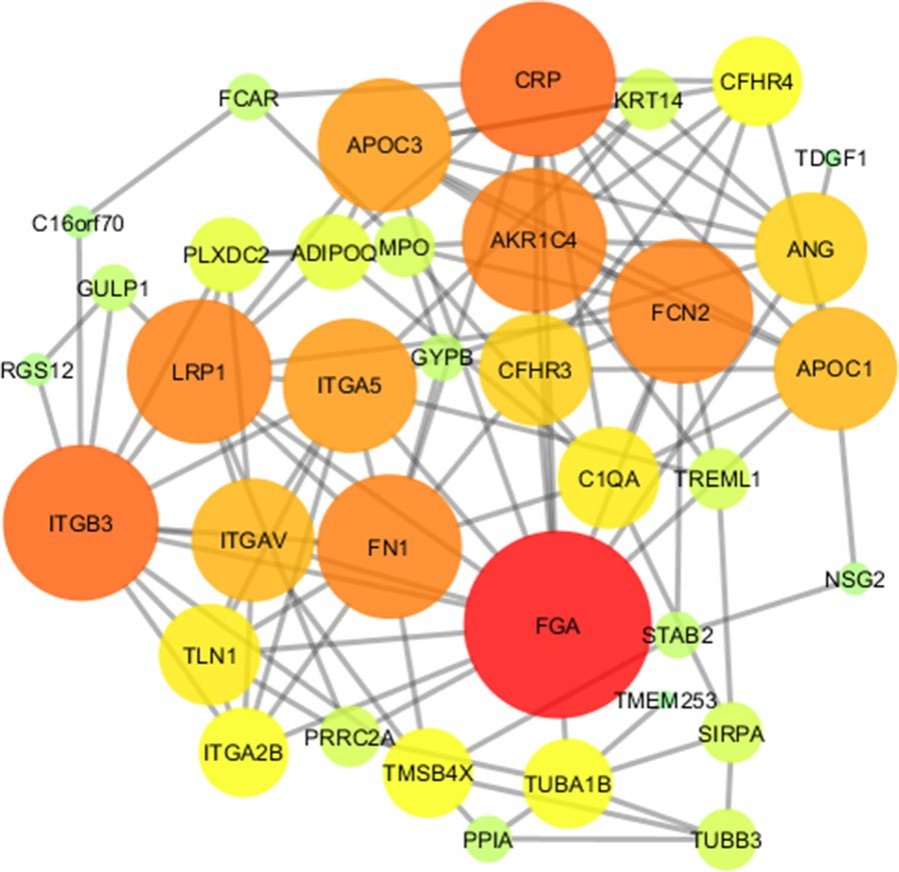
PPI network. The analysis included proteins that showed a significantly differential abundance between ML and HC groups. Proteins are represented as nodes while interactions appear as link. The quantity of link relates to the strength of the interaction associations (In the colors used in the figure, there is a color gradient from red to green that red color was used to high degree proteins in the PPI, green color was used to low degree proteins in the PPI)

**Fig. 4:**
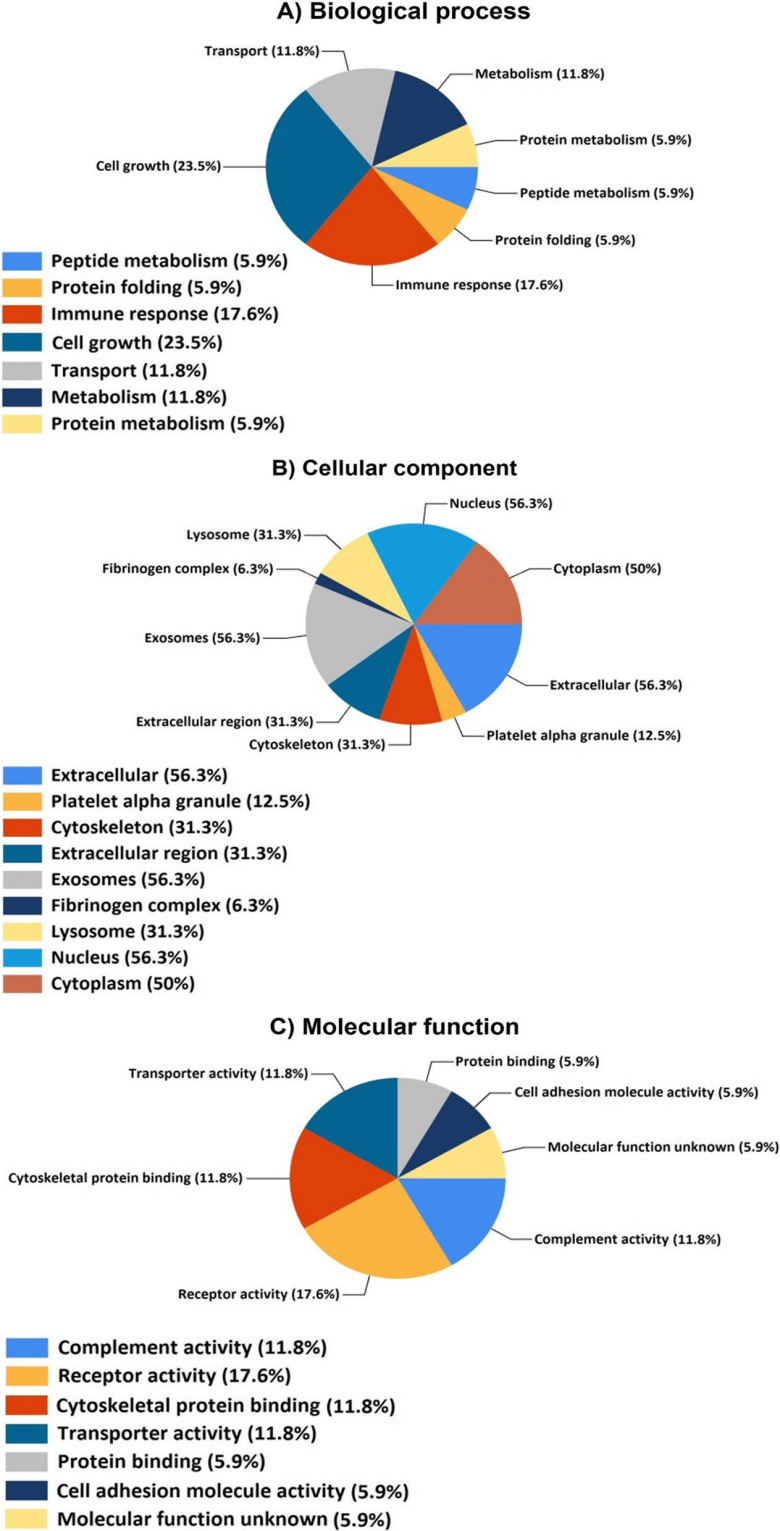
GO enrichment analysis in BP, CC and MF. Note:** A** refers to biological process,** B** refers to cellular component,** C** refers to molecular function

The unweighted PPI network was constructed using GeneMANIA tool by 20 proteins for assay GO enrichment analysis (up and down) (Fig. 3). Furthermore, more explanation details about the PPI network were provided in Additional file 2. GO enrichment analysis in the cellular component showed that the proteins were massively enriched in extracellular and exosomes (56.3%), followed by in the cytoskeleton (31.3%). In molecular function analysis, the largest proportions of proteins were involved in receptor activity (17.6%) and in cytoskeletal protein binding, and complement activity (11.8%). In biological process analysis, proteins were involved in response to cell growth (23.5%), immune response (17.6%), and protein folding, peptide metabolism (5.9%) (Fig. 4). Additionally, KEGG pathway enrichment analysis revealed a total of 11 primary pathways that were strongly connected to proteins with various expressions (Table 6).

**Table 6:** KEGG pathways in protein dataset

KEGG Pathway Term	Observed	Background	*p* value
description	Gene(n)	Gene (n)	Rate
IFN-gamma pathway	5	1297	0.03
TRAIL signaling pathway	5	1293	0.03
Arf6 signaling events	5	1292	0.03
Sphingosine 1-phosphate (S1P) pathway	5	1285	0.03
LKB1 signaling events	5	1304	0.03
Glypican pathway	5	1293	0.03
IL5-mediated signaling events	5	1285	0.03
Nectin adhesion pathway	5	29	0.04
VEGF and VEGFR signaling network	5	1308	0.03
Integrin family cell surface interactions	5	1285	0.03
Hemostasis	4	1285	0.03

## Discussion

During any type of disease, the proteins are the most functional and effective molecules. Understanding protein relationships gives insight into previously unknown aspects of the pathophysiology of hazardous substance interactions with human body. Proteomic studies of serum with system biology approach are the best ways to detect changes in the pathophysiology in cells [35–37]. COPD is a chronic inflammatory illness that shares many of the same signs and symptoms as people who were exposed to SM gas; with the exception that ML is not a progressive condition. Based on analysis of demographic data, the independence and randomization of patients as well as control patients were shown. All patients had notable air trapped in their lungs which is the principal symptom of SM-induced damage known as ML. In the ML, the majority of these significantly altered proteins, according to GO analysis, were involved in inflammatory processes and immune system-associated proteins. Cell adhesion and integrity, as well as cell interaction with the extracellular matrix, and present in pathways, which represent a major roadblock in tissue regeneration process in ML. There were some proteomic studies on SM exposure serum or plasma, but none have used high via the techniques. Haptoglobin isoforms and Amyloid A1 were previously identified as two of the most commonly changed proteins in ML individuals' plasma and BAL fluid [35–38]. Another study found changes in a wide range of polymorph nuclear cells (PMNCs) proteins, including S100 family members, antioxidant proteins, Serpin B1, and Cronin 1A. An imbalance in protease/anti-protease activity was also observed in PMNCs results in an uncontrolled and unjustifiable response to stimuli and signals from wounded organs [11]. In general, the elevation of antioxidant and inflammatory proteins, as well as the downregulation of protective and preventive proteins, suggests a continuing process of inflammation and remodeling in ML patients. However, several COPD studies have found plasma proteins differently expressed than the control [39–42]. A new element of continuing pathophysiology of SM-exposed patients was presented. As expected, most differentially expressed proteins are involved in healing and remodeling processes. However, the discovery of several intercellular proteins, such as Thymosin beta-4, which not only have a structural role in cellular components, but also play a part in extracellular matrix(ECM) regeneration and remodeling in healing tissues, casts doubt on earlier assumptions. Thymosin beta-4 is a regenerative peptide linked to wound healing, angiogenesis, cardiomyocyte migration, repair, anti-fibrotic [43–47]. It can regulate the actin polymerization process and act as an anti-inflammatory in human fluids [48, 49]. Compared to HC, high overexpression levels show that tissue regeneration and cell migration are still present in wide areas of wounded tissues. Upregulation of proteins, such as CFHR3, CRP, IGLV3-25, IGLV3-21, IGKV4-1, MPO, FCN2 and TREML1, indicates an activated immune system and an inflammatory or pro-inflammatory response by tissue cells, as well as other body systems in order to orchestrate a coordinated response against cell injury. All of proteins play a distinct role in the cleansing of tissue by cell death, the development of a basal niche for new cell growth, and attraction of effective cells in healing process, which includes angiogenesis and ECM remodeling. Specific proteins, such as TLN1, FGA, ITGB3, PPIA and TUBA1B would be required to regulate cell adhesion and spread following the establishment of conditions for cells to proliferate. Cells may attach to modified ECM and freshly replaced neighbor cells thanks to these substances' ability to modify and control cell connections and integrity. The majority of the proteins that were downregulated are involved in lipid metabolism and circulation in the human body, including APOCI, APOCIII, and adiponectin. Obesity and metabolic syndrome are both linked to these proteins. Reduced levels of these proteins contribute to higher cholesterol and triglyceride levels in the liver and fatty tissue, which can lead to cardiovascular disease. Adiponectin deficiency results in inadequate mitochondrial energy output [50]. It increases insulin sensitivity by inhibiting gluconeogenesis and promoting fatty acid oxidation [51]. The pathogenesis of ML shows a modified continual healing procedure in which cells strive to proliferate and duplicate normal tissue layers, but the lack of a bed to mount the layers leads to constant cell death and destruction of secreted proteins, as well as tissue layers. The presence of defined proteins in the serum indicates that there is ongoing cell damage and that the cells are unable to heal the damaged organ. As previously stated, the presence of many repairs and balancing components in discovered proteins shows that cells always strive to produce suitable conditions for regenerating previously balanced and normal conditions. The absence or the downregulation of critical signals, such as PLXDC2 and fundamental extracellular matrix proteins such as KRT14, as well as the overexpression of proinflammatory proteins, results in an uncontrollable cycle of tissue layer repair and destruction. Histopathological studies, as well as particular immunofluorescent staining of ECM and cytoskeletal elements of biopsies taken from exposed patients, are highly recommended to understand the process and unravel the intricacy of the phenomena. Considering research ethics difficulties, age, and severe to moderate patient conditions, this strategy was not feasible. At the same time as this study was conducted on serum, the proteome analysis of skin biopsy and eye tear samples of ML and HC subjects was performed with the same method and number of samples [52, 53].

## Conclusion

SM is an oxidative chemical which quickly reacts with various cell components.

In repairing the damaged parts of lung, epithelial cells, macrophages, and fibroblasts are activated in the damaged area. After decades of exposure to SM, there was a significant rise in the presence of key proteins which secrete in healing cells. The overexpression of adhesion and structural proteins such ITGB3 and TUBA1B cause the damaged cells to try harder to attach to ECM and move to form new tissues. The downregulation of KRT14, which is found in cell–cell adhesions and is a crucial component for cell migration and healing. In ML patients, it appears that the tissue repair cycles are disrupted. Although this event primes cell regeneration and proliferation, it also coordinates the immune system's pro-inflammatory components to set the stage for optimal tissue repair, resulting in a steady stream of inflammatory indicators.

## Supplementary Information

**Additional file 1**. The list of differentially abundant proteins.

**Additional file 2**. The details of the PPI network in the Cytoscape tool.

## Data Availability

The data which support this study's findings are available from the corresponding author upon reasonable request.

## Abbreviations

SM: Sulphur Mustard
ML: Mustard Lung
TMT: Tandem Mass Tag
EA: Enrichment analysis
COPD: Chronic pulmonary obstruction
BAL: Broncho alveolar lavage
DAPs: Differentially abundant proteins
GO: Gene ontology
ATS: American Thoracic Society
PFT: Pulmonary function testing
CO: Carbon monoxide
FEV1% Pred: Predicted forced expiratory volume in one second
FVE: Forced Vital Capacity
RT: Room temperature
APAF: Australian Proteome Analysis Facility
PBS: Phosphate buffer saline buffer
DTT: Dithiothreitol
IAA: Ioadoacetamid
ACN: Anhydrous acetonitrile
PPIs: Protein–protein interactions
KEGG: Kyoto Encyclopedia of Genes and Genomes
FC: Fold change
